# Process Strategies Enabling Selective Polymer Valorization from Textile Fiber Blends

**DOI:** 10.3390/ma19102100

**Published:** 2026-05-16

**Authors:** Diana Smarandache, Bruno Godinho, Marina Matos, Susana C. Pinto, Cătălina Ionescu, Nicoleta Cioateră, Artur Ferreira, Nuno Gama

**Affiliations:** 1 Department of Chemistry, Faculty of Sciences, University of Craiova, 107i Calea București Street, 200512 Craiova, Dolj, Romaniacatalinagurgui@yahoo.co.uk (C.I.); nicoletacioatera@yahoo.com (N.C.); 2 CICECO—Aveiro Institute of Materials, Department of Chemistry, University of Aveiro, Campus Universitário Santiago, 3810-193 Aveiro, Portugal; bruno.godinho@ua.pt (B.G.); marina.matos@ua.pt (M.M.); scpinto@ua.pt (S.C.P.); 3 CICECO—Aveiro Institute of Materials, Escola Superior de Tecnologia e Gestão de Águeda—Rua Comandante Pinho e Freitas, No 28, 3750-127 Águeda, Portugal; artur.ferreira@ua.pt

**Keywords:** textile waste, selective chemical recycling, mixed fiber valorization, circular economy

## Abstract

**Highlights:**

- Selective chemical recycling of mixed textile waste via polymer-specific routes.
- Recovery of acrylic, nylon, polyester and cotton fibers through tailored processes.
- Incorporation of recycled products into new polymer matrices.
- Demonstration of the feasibility of closed-loop textile-to-polymer valorization strategies.

**Abstract:**

The increasing complexity of textile waste, particularly blended fibers, represents a major challenge for conventional recycling approaches. This study proposes a selective valorization strategy for mixed textile waste streams by applying tailored chemical recycling routes to individual fiber type. Preliminary tests identified suitable methodologies for each fiber type: dissolution–precipitation for acrylic (poly(acrylonitrile)—PAN), acidolysis for nylon, glycolysis for polyester (PeS) and acetylation for cotton. Structural characterization confirmed that the incorporation of recycled products did not significantly change the chemical structure or crystallinity of the resulting materials. Furthermore, thermal analysis revealed comparable or slightly improved thermal stability in most recycled systems. Additionally, mechanical performance was observed to vary depending on the polymer type. Recycled acrylic and cellulose acetate showed reduced ductility, while nylon exhibited increased stiffness due to possible recrystallization effects. In contrast, PeS displayed enhanced elongation at break, suggesting increased chain mobility or plasticization effects. Overall, the results demonstrate that selective chemical valorization is a promising route for the efficient recycling of complex textile waste, enabling the recovery of high-quality materials with retained functional properties.

## 1. Introduction

Global textile production has experienced rapid growth in recent decades, largely driven by fast fashion, which prioritizes mass production [1], having the global fiber production increased from 51.4 million tons in 2000 to 108.3 million tons in 2020, being projected to reach 300 million tons by 2050 [2]. This rapid expansion has reinforced a predominantly linear economic model based on “produce–consume–discard”, significantly increasing the generation of post-consumer textile waste. In response, the textile sector is progressively shifting toward a circular economy paradigm, where textile waste is increasingly considered a valuable raw material rather than a disposable residue.

The textile industry uses fibers that originate from natural sources including plant-based fibers such as cotton, hemp, flax bamboo; animal-derived fibers like wool and silk; mineral fibers such as asbestos; and synthetic fibers including polyester (PeS), nylon, and acrylics elastane. Cotton and polyethylene terephthalate (PET) dominate the fiber usage, together accounting for roughly 88% of total output, with PeS alone representing 57% in 2023. The increasing diversity and complexity of textile compositions, particularly the widespread use of blended fibers, coatings, dyes and additives, have considerably complicated recycling and material recovery processes.

PET is a semi-crystalline PeS containing ester linkages and is widely used in fiber blends. In turn, cotton, composed of D-glucose polymer chains, is strong, flexible and hydrophilic [3]. Additionally, polyacrylonitrile (PAN) is a type of acrylic which is highly crystalline and chemically resistant. Another material used in fiber production is nylon, which consists of linear polyamide (PA) chains that impart strength and elasticity. The distinct chemical structures and physicochemical properties of these fibers strongly influence their recyclability and determine the suitability of different recycling pathways.

As mentioned, the expansion of the textile sector has increased the environmental pressures due to excessive water use, chemical pollution, greenhouse gas emissions and the generation of over 92 million tons of waste annually. Notably, approximately 75–85% of post-consumer textile waste is either landfilled or incinerated, contributing to chemical contamination and microplastic pollution [4]. This is mainly due to the fact that the current recycling approaches face significant limitations, because fiber properties directly influence recyclability, and therefore less than 1% of textile fibers are recycled, emphasizing a major gap in sustainable material recovery [5]. Increasing regulatory pressure, particularly within the EU, is promoting separate textile waste collection, extended producer responsibility schemes and eco-design strategies aimed at improving recyclability and reducing environmental impacts.

Mechanical recycling methods, such as shredding, carding and re-spinning, are simple and cost-effective but degrade fiber quality and perform poorly on blended textiles. Chemical recycling techniques including hydrolysis, glycolysis and methanolysis can recover monomers from synthetic fibers like PET and nylon, requiring high energy and hazardous chemicals, whilst also being sensitive to contamination [6]. Solvent-based approaches such as dissolution–precipitation processes offer another avenue, enabling selective dissolution and recovery of valuable polymers, and they have been used to successfully recover a variety of polymers [7]. Thermal treatments such as incineration, pyrolysis and melt re-extrusion allow energy or material recovery but may release pollutants and degrade natural fibers. In recent years, chemical and solvent-based recycling technologies have gained increasing attention because they enable the recovery of higher-quality materials with properties closer to virgin polymers, particularly for synthetic fibers such as PET and nylon.

Although recycling methods for individual textile fibers are documented, valorizing mixed-fiber textiles is challenging due to the complexity of fiber blends and their interactions. To address this, the present work proposes an integrated, selective recycling strategy for mixed textile waste. This approach uses sequential, controlled treatments tailored to the chemical structure and reactivity of each fiber, rather than non-selective bulk processing. By combining dissolution–precipitation techniques with depolymerization and modification reactions, this study establishes a potential, adaptable and scalable framework for recovering high-value polymer building blocks from complex textile streams. Such an approach aligns with the current paradigm of textile recycling, which aims not only to divert waste from landfill and incineration, but also to maintain materials within the production cycle for as long as possible through high-value valorization strategies.

## 2. Materials and Methods

### 2.1. Materials

Textile fibers were obtained from a local store. In the dissolution–precipitation experiments, textile fibers were dissolved in dimethyl sulfoxide (DMSO) supplied by Fisher. After the dissolution–precipitation process, the recovered products were blended with poly(methyl methacrylate) (PMMA) powder (Mw = 350,000; Tg = 122.0 °C) supplied by Sigma–Aldrich. In the acidolysis reactions, textile fibers were reacted with adipic acid obtained from Sigma–Aldrich. The recovered products of the acidolysis reactions were subsequently mixed with 6-aminohexanoic acid, also supplied by Sigma–Aldrich, to produce new nylon materials. In glycolysis reactions, ethylene glycol and glycerol of pharmaceutical grade, supplied by Sigma–Aldrich, were used as glycolysis agents, with zinc acetate (Sigma–Aldrich) used as the catalyst. The glycolysis products were then used to synthesize new PeS using sebacic acid (99% purity, Sigma–Aldrich). In the acetylation reactions, acetic anhydride (≥98.0%), glacial acetic acid and sulphuric acid (ACS reagent, 95.0–98.0%), all supplied by Sigma–Aldrich, were used. The acetylated products were subsequently blended with cellulose acetate pellets (average Mn ~30,000, Sigma–Aldrich) to produce filaments.

### 2.2. Valorization of Fibers Mixture

In the valorization methodology, mixtures consisting of equal masses of PAN, nylon, PET and cotton fibers were used. The dissolution–precipitation experiments were carried accordingly with [8], where textile fibers were dissolved in DMSO in a glass round bottom balloon at 25 °C, using a solvent-to-polymer ratio (v/wt) of 50, for 2 h, under magnetic stirring at 150 rpm. Acidolysis reactions were carried out accordingly with [9] by reacting textile fibers with adipic acid at a mass ratio of 1.0 (wt acid/wt fiber), in a glass round bottom balloon, at 200 °C, for 5 h, under a nitrogen atmosphere and magnetic stirring at 150 rpm. Glycolysis reactions were performed accordingly with [10] using a solvent-to-fiber mass ratio of 12.0 (wt solvent/wt fiber), with an ethylene glycol/glycerol mass ratio of 1:1 (wt/wt) and using zinc acetate as a catalyst at 0.5 wt% relative to the total mass of fiber and solvent. The reactions were carried out in a glass round bottom balloon at 195 °C, for 5 h, under a nitrogen atmosphere and magnetic stirring at 150 rpm. Acetylation reactions were conducted accordingly with [11] by reacting the fibers with acetic anhydride at a mass ratio of 4.0 (wt anhydride/wt fiber), acetic acid at a mass ratio of 6.0 (wt acid/wt fiber) and sulphuric acid used as a catalyst at 0.5 wt% relative to the fiber mass. The reactions were carried out in a glass round bottom balloon at 80 °C, for 10 min, under a nitrogen atmosphere and magnetic stirring at 150 rpm. Please note that, after each recovery step, no purification step was carried out, with the recycled products used as such in the production of new materials.

### 2.3. Preparation of New Materials

In the extrusion of PMMA filaments (neat PMMA and PMMA blended with 10 wt% of products obtained from the dissolution–precipitation step), they were extruded using a Felfil Evo Colours extruder operating at 190 °C and 9 rpm.

In the synthesis of nylon materials, 6-aminohexanoic acid was blended with recycled products obtained from the acidolysis step (0 wt% or 10 wt% relative to the total monomer content) and polymerized in a round-bottom flask at 205 °C for 15 min. The resulting polymer was subsequently cast into a rectangular mold.

The synthesis of new PeS was carried out using an ethylene glycol/glycerol ratio of 1:1 (wt/wt) and sebacic acid, corresponding to an overall equimolar ratio (1:1) of hydroxyl groups to carboxylic acid groups. Pre-polymerizations were conducted up to 50% of esterification, at 150 °C in a PARR reactor equipped with a 300 mL stainless-steel vessel, under a nitrogen atmosphere. Subsequently, the polymerization step was performed in a VT 6025 vacuum oven (Thermo Scientific) coupled to an ILMVAC MP 601 T vacuum pump (ultimate pressure < 1 mbar), at 150 °C, under vacuum.

In the extrusion of cellulose acetate filaments (neat cellulose acetate and cellulose acetate blended with 10 wt% of products obtained from the acetylation step), they were extruded using a Felfil Evo Colours extruder at 235 °C and 3 rpm.

### 2.4. Characterization

Recycled products, as well as both neat and materials incorporated with recycled products, were characterized by Fourier-transform infrared spectroscopy (FTIR), solid-state ^13^C Cross-Polarization Magic Angle Spinning Nuclear Magnetic Resonance (^13^C CPMAS NMR), X-ray diffraction (XRD), mechanical properties, Differential Scanning Calorimetry (DSC) or Thermogravimetric Analysis (TGA).

The FTIR spectra were collected on a Perkin Elmer FTIR System Spectrum BX Spectrometer equipped with a single horizontal Golden Gate ATR cell. All data were recorded at room temperature, in the range 4000 to 600 cm^−1^ by accumulating 32 scans with a resolution of 4 cm^−1^.

^13^C CPMAS NMR spectra were recorded on a Bruker Avance 400 spectrometer. Samples were packed into a zirconia’s rotor sealed with Kel-FTM caps and spun at 12 kHz. Acquisition parameters were as follows: ca 7000 scans with a 90 proton pulse, a crosspolarization contact time of 1 ms and a recovery delay of 2.5 s.

XRD patterns were collected using a Philips X’pert MPD diffractometer (Eindhoven, The Netherlands) using Cu Kα radiation. The scattered radiation was detected in the angular range from 5° to 50° (2θ).

Tensile tests were conducted on a Hegewald & Peschke universal testing machine Inspekt solo, equipped with a 2.5 kN load cell, in accordance with DIN EN ISO 527 [12]. Tensile tests were performed at a speed of 10 mm·min^−1^ up to the breaking point.

DSC analyses were carried out using a Netzsch DSC 204F1 Phoenix, from −50 °C up to 200 °C, at a heating rate of 10 °C·min^−1^.

TGA was performed using a SETSYS Evolution 1750 thermogravimetric analyzer (Setaram) from room temperature up to 800 °C, at a heating rate of 10 °C·min^−1^ and under nitrogen flux (200 mL·min^−1^).

## 3. Preliminary Valorization Essays

The strategy for valorizing the textile mixture began by identifying the most suitable valorization method for each fiber type.

PAN is a type of acrylic polymer commonly used as textile fiber, having a global production of ~1.6 Mt in 2023, yet only about 0.6% is recycled. These fibers are non-biodegradable, persisting in the environment and contributing to waste accumulation and microplastic formation. This is mainly because its recycling is challenging because commercial PAN contains 15–20% comonomers, different molecular weights, and additives that introduce defects and degrade mechanical properties [13]. In fact, mechanical recycling methods are poorly suited for acrylics due to this composition complexity [13]. However, chemical recycling via dissolution–precipitation using specific solvents, such as DMSO, can be a viable alternative [13,14].

Nylon 6 and Nylon 6,6 belong to the class of PA and are among the most widely used crystalline engineering thermoplastics, with important applications such as textile fibers [15]. However, nylon exhibits low biodegradability, causing residues which persist in the environment if not properly disposed of [16]. Recycling methods for nylon waste can be classified as either mechanical or chemical. Recently, a novel chemical recycling approach has been proposed, utilizing dicarboxylic acid in a process known as acidolysis [9]. The results showed that incorporating recycled products into new nylon materials did not alter their chemical or crystalline structure. The sustainability of this novel recycling route was evaluated using a life cycle assessment (LCA) and its advantage was highlighted when compared with the conventional chemical recycling method for nylon, which involves hydrolysis with HCl.

PET is a versatile thermoplastic polymer widely used in the packaging and textile industries. However, growing concerns over the non-biodegradability of PET waste and the urgent need for sustainable practices have made its recycling essential. As a result, PET is one of the most extensively studied polymers for recycling, which can be achieved through physical, chemical or biological approaches [10]. Among these, glycolysis is likely the most used method [17].

Cotton is one of the most widely used raw materials in the textile industry and, after PeS, the most consumed fiber, making waste cotton one of the most prevalent forms of textile waste. Recycling waste cotton is challenging because cotton is often blended with other natural or synthetic fibers [18]. Additionally, recycled cotton fibers are typically of lower quality, limiting their suitability for clothing production. Current recycling methods for waste cotton primarily include mechanical and chemical biological approaches [19]. Beyond recycling, this type of cellulose-based material can be transformed into value-added products. Notably, cellulose derivatives play a key role in industry, as cotton can be chemically modified such as through esterification to produce cellulose acetate, which is one of the most important commercial cellulose derivatives due to its broad range of applications [20].

Based on the literature, a range of different methods capable of valorizing each type of textile was selected. Table 1 lists the recycling strategies used, highlighting their suitability for the valorization of each textile fiber type.

Based on the results presented in Table 1, the reactivity of textile fibers strongly depends on the type of methodology used. Acrylic fibers could only be valorized through dissolution, in which a 100% liquid phase containing the dissolved polymer was obtained. After solvent removal by distillation, the polymer was successfully recovered. Furthermore, acrylic was the only textile fiber that could be dissolved in DMSO. In contrast, nylon fibers proved to be susceptible to both acidolysis and glycolysis, which cleave their polymer chains, resulting in a viscous recycled product absent of solid particles. Similarly, although PET fibers were resistant to acidolysis, they were effectively depolymerized through glycolysis. In the chemical recycling of both nylon and PET, the polymers were converted into recycled products that can be used to synthesize new materials. Finally, cotton, being a cellulose-based polymer, did not react under dissolution–precipitation, acidolysis or glycolysis conditions, but it converted into cellulose acetate through acetylation. This information is essential for understanding how different polymers can be valorized.

**Figure 1:**
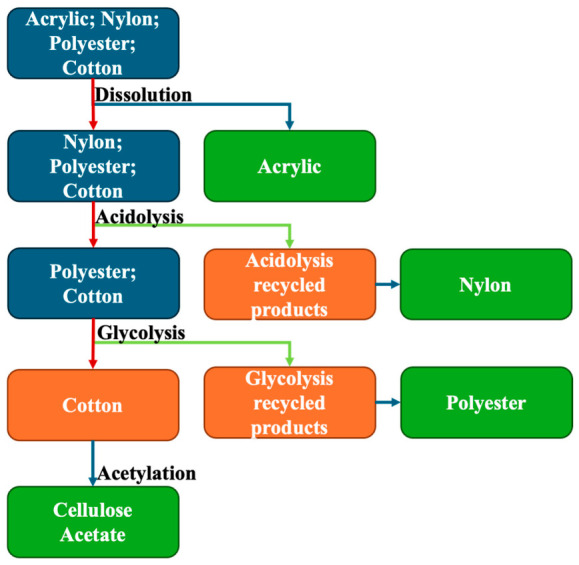
Flowchart of the selective textile fiber valorization from the mixture of textile fibers.

Based on the preliminary tests, a selective valorization methodology of each textile fiber type from a mixed textile waste stream was established, as illustrated in Figure 1. The objective is to valorize each textile fiber type individually, thereby avoiding the formation of complex mixtures.

## 4. Valorization of a Mixture of Fibers

As previously mentioned, the selective valorization of the textile fiber mixture followed the flowchart depicted in Figure 1. After the recycling, each type of recycled product was used to produce new materials: (i) PMMA was extruded either as neat PMMA or blended with 10 wt% of recovered acrylic products; (ii) nylon materials were synthesized incorporating acidolysis recycled products at 0 wt% or 10 wt% relative to the total monomer content; (iii) PeS materials were synthesized including glycolysis recycled products at 0 wt% or 10 wt% relative to the total monomer content; and (iv) cellulose acetate materials were extruded, either as pure cellulose acetate or blended with 10 wt% of acetylated products. The mechanical properties of the resulting materials are summarized in Table 2.

### 4.1. Dissolution–Precipitation of Acrylic Fibers

**Figure 2:**
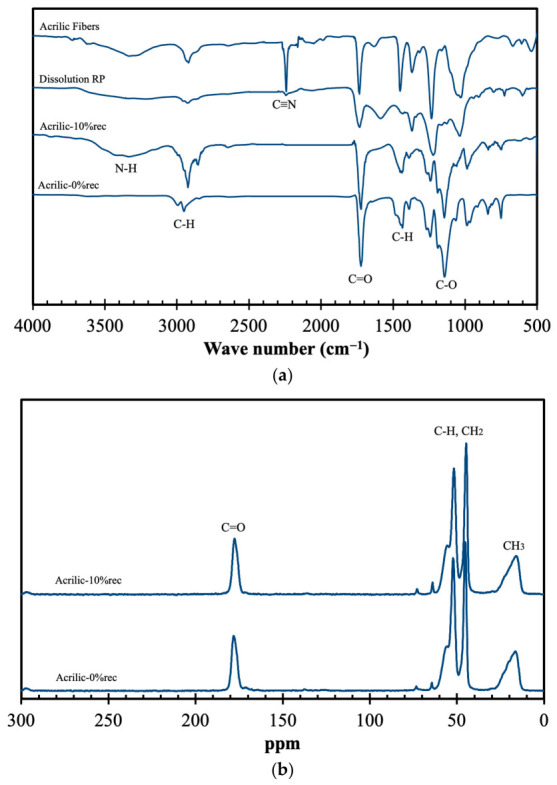

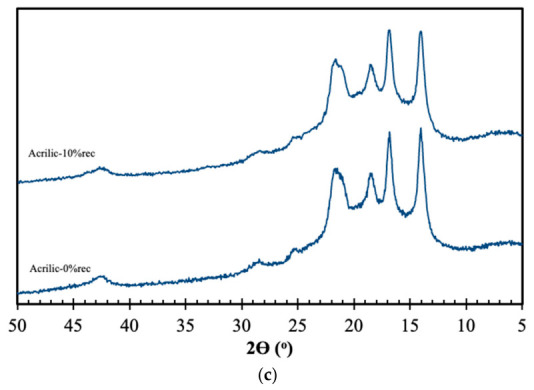
Spectroscopic analyses of original acrylic fibers, products obtained via dissolution–precipitation (RP), both virgin acrylic and acrylic containing 10% recycled material: FTIR (**a**), ^13^C CPMAS (**b**) and XRD (**c**).

Following the flowchart presented in Figure 1, the selective valorization of textile fibers from a mixed textile stream started with the recovery of acrylic (PAN) fibers through dissolution–precipitation. To assess the viability of this method, the chemical composition of the original fiber, the recovered polymer and its subsequent incorporation into new acrylic products were evaluated by spectroscopic analyses (see Figure 2).

The FTIR spectra shown in Figure 2a exhibit the typical profile of acrylic polymers, with characteristic absorption bands corresponding to C≡N, C=O, C–O and C–H bonds [21]. A broad band around 3500 cm^−1^ is observed and attributed to N–H stretching vibrations, while the absorption bands in the 3030–2830 cm^−1^ region are associated with C–H stretching. A sharp peak at approximately 2240 cm^−1^ is clearly visible and corresponds to the presence of nitrile (C≡N) groups. The absorption band around 1730 cm^−1^ is related to C=O stretching vibrations, whereas the peak at about 1450 cm^−1^ can be ascribed to the symmetric bending vibrations of C–H bonds in the hydrocarbon backbone. Finally, the peak observed near 1050 cm^−1^ is attributed to C–O stretching vibrations. Interestingly, despite having a similar profile to the original fiber, the recycled product does not exhibit the peak centered at 2240 cm^−1^. This suggests partial chemical modification of PAN structure during previous processing and manufacturing steps. In particular, nitrile groups can undergo hydrolysis or thermo-oxidative reactions, leading to the formation of amide (–CONH_2_) and carboxyl (–COOH) groups [22]. Indeed, a new peak at 1600 cm^−1^ is observed in the recycled product, which can be attributed to C=O bonds. In contrast, when comparing the new acrylic materials and the 10% composites incorporating recycled products, no significant differences are noticeable, apart from the appearance of the new band at 3500 cm^−1^ due to N–H stretching vibrations introduced by the recovered recycled product. The formation of these oxygen-containing functional groups may increase the polarity and intermolecular interactions within the material, potentially affecting properties such as thermal stability, compatibility with the matrix and mechanical performance of the final recycled product, as it will be discussed later.

Analyzing the ^13^C CPMAS spectra presented in Figure 2b, it can be observed that both acrylic samples exhibit an identical profile. The peak between 10 and 20 ppm is attributed to the CH_3_ moieties bonded to quaternary carbons and the quaternary carbons themselves, while the peaks at 45 and 52 ppm correspond to the C–H and CH_2_ moieties [23]. Finally, the peak at 178 ppm is assigned to the C=O group. Comparing the ^13^C CPMAS spectra of neat PMMA and PMMA incorporated with recycled products, no differences are detected, indicating that both samples possess an identical chemical structure; thus, the incorporation of recycled products does not induce any detectable changes.

Analysis of the XRD pattern of the acrylic materials shown in Figure 2c reveals four diffraction peaks, which were not expected, since PMMA is typically an amorphous polymer [24,25]. The presence of such peaks can be attributed to the extrusion process, which may reorganize the crystalline structure of the polymer. Nonetheless, from Figure 2c it can be seen that incorporating 10% recycled material into the neat acrylic does not significantly alter the XRD patterns, suggesting that the acrylic maintains a similar degree of crystallinity.

Regarding the mechanical properties, from Table 2, it can be seen that the incorporation of recycled acrylic products into PMMA results in a decrease in stiffness since the neat material exhibited a tensile strength of 6328.9 ± 225.4 kPa and after the incorporation of recycled products, the tensile strength decreased to 3756.8 ± 351.1 kPa. Additionally, the Young’s modulus dropped from 532.1 MPa to 473.7 MPa (~11%), while the elongation at break decreases significantly from 50.6% to 37.8% (~25%). These observations suggest that the recycling process may induce chain scission or molecular weight reduction, and microstructural defects such as voids or incomplete fusion could also contribute to the increased brittleness. Nonetheless, it should be noted that recycled acrylic products are derived from PAN and are incorporated into PMMA, so the differences in mechanical properties can be attributed to differences in the material’s intrinsic nature.

**Figure 3:**
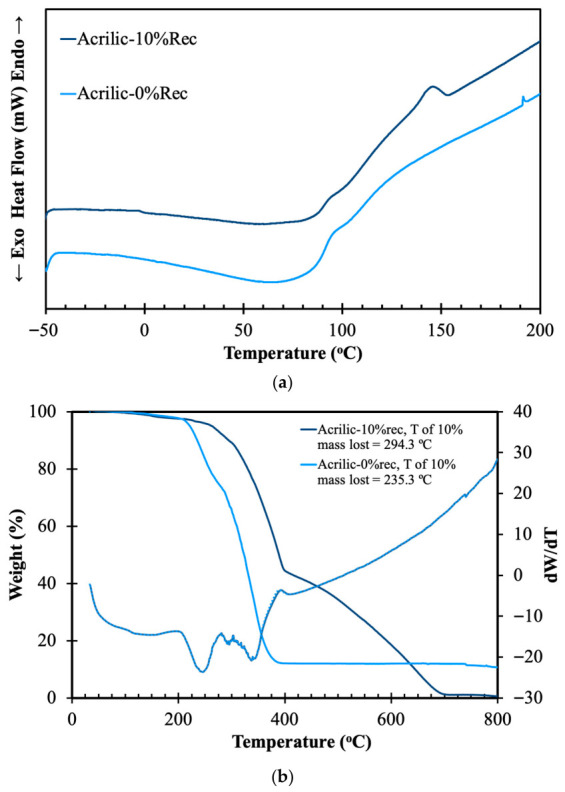
DSC (**a**) and TGA (**b**) of virgin acrylic and acrylic containing 10% recycled material.

Additionally, the thermal profile of the materials was analyzed, and the results are presented in Figure 3.

From Figure 3a, it can be noticed that the acrylic samples exhibit broad thermal transitions without a distinct melting peak. The virgin acrylic sample (0% Rec) shows a relatively stable baseline from −50 °C to approximately 80 °C, followed by a gradual endothermic transition extending toward higher temperatures. This behavior is associated mainly with glass transition and molecular relaxation processes occurring within the amorphous regions of the polymer. The acrylic sample containing 10% recycled fibers presents an endothermic response and a more pronounced thermal transition between approximately 100 °C and 160 °C. The intensity of the thermal event suggests that recycled acrylic fibers promote changes in chain mobility and structural organization.

In Figure 3b, the TGA curves of virgin acrylic and acrylic containing 10% recycled material are presented. Both acrylic samples exhibit a two-step degradation behavior. The Acrylic-0%rec sample (PMMA-based matrix) shows a relatively simpler and less resolved degradation profile, consistent with the thermal decomposition of PMMA. In contrast, the Acrylic-10% recycled sample exhibits a more defined two-step degradation pattern. This additional feature is attributed to the presence of the recycled fraction derived from PAN-based material incorporated into the PMMA matrix. Additionally, the Acrylic-0% recycled sample loses 10% of its mass at 235 °C, while the Acrylic-10% recycled sample reaches 10% mass loss at 294 °C, indicating higher thermal stability. Examining the thermogram of the Acrylic-0% recycled sample, a degradation step centered at 350 °C can be seen, associated with the decomposition of monomeric methyl methacrylate and CO_2_, typical of PMMA [26]. In contrast, looking at the Acrylic-10% sample in more detail, a weight loss is observed between 225 °C and 400 °C, being attributed to dehydrogenation and the release of H_2_, while the following degradation step occurring beyond 400°C is due to the partial volatilization of ammonia and hydrogen cyanide. These observations are in agreement with the results reported by Basiouny et al. [27].

### 4.2. Acidolysis of Nylon Fibers

**Figure 4:**
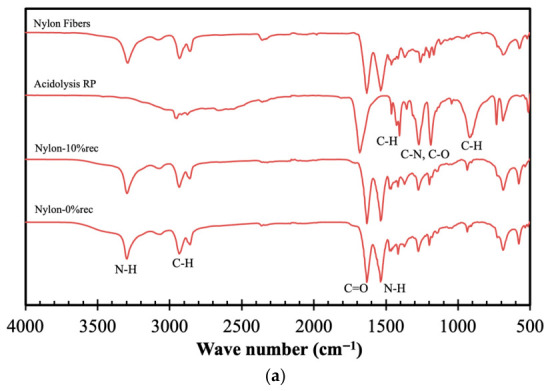

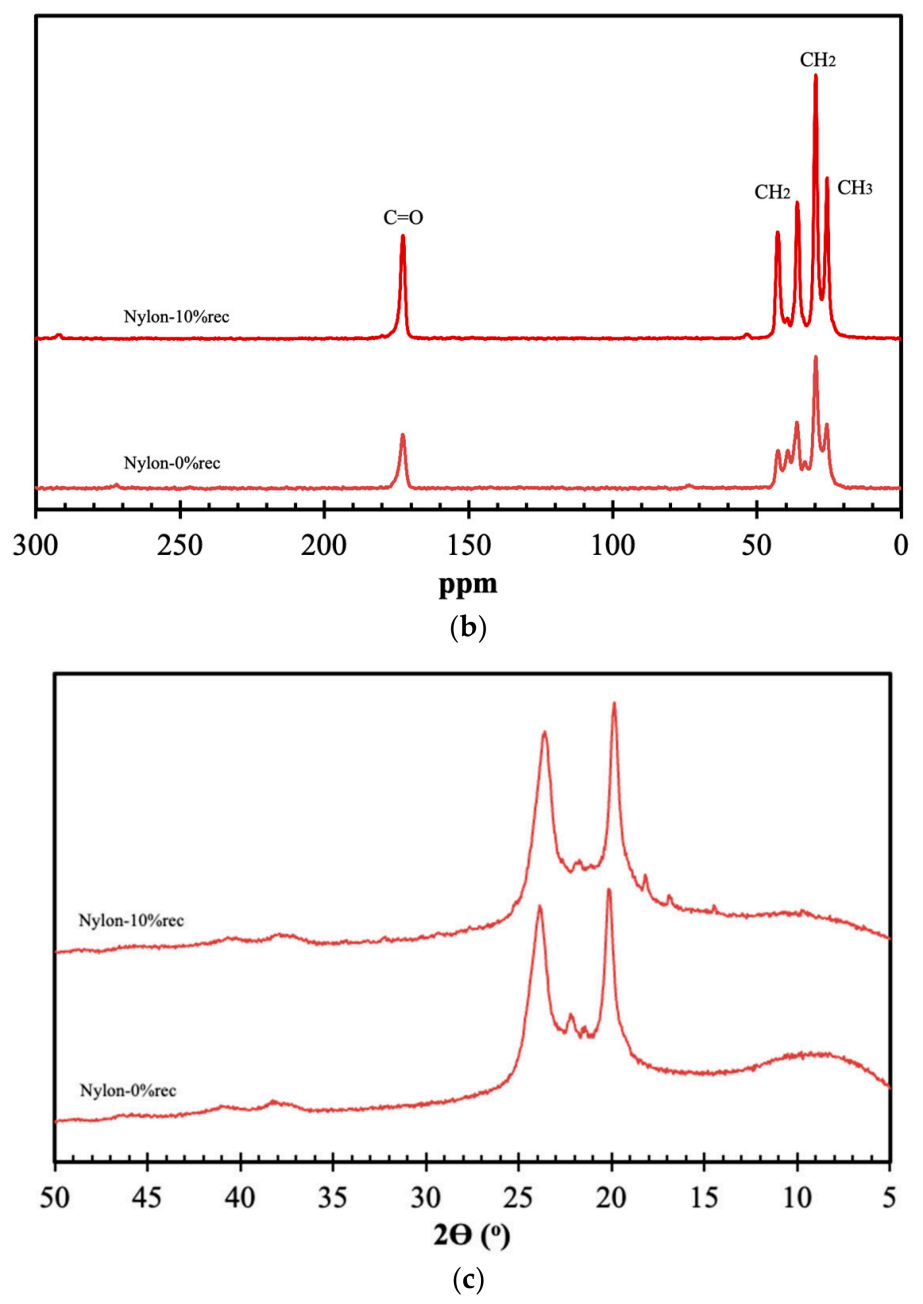
Spectroscopic analyses of original nylon fibers, products obtained via acidolysis of nylon, both synthetized nylon and nylon containing 10% recycled material: FTIR (**a**), ^13^C CPMAS (**b**) and XRD (**c**).

Following the flowchart presented in Figure 1, the following selective valorization step was the acidolysis of nylon fibers, with the spectroscopic analyses of the ensuing materials presented in Figure 4.

The FTIR results presented in Figure 4a show that the original fibers exhibit the typical spectra of nylon, while the recycled products display prominent peaks primarily associated with the raw materials (nylon and adipic acid) [9]. The peak at 3300 cm^−1^ corresponds to N–H functional groups, while the peaks at 2930 and 2850 cm^−1^ are attributed to methylene groups (CH_2_). The peak at 1685 cm^−1^ arises from the stretching vibration of C=O bonds in adipic acid. The peak around 1640 cm^−1^ is assigned to amide I, characterized by C=O stretching; the peak at 1540 cm^−1^ corresponds to amide II, associated with N–H bending in PA. C–H bonds are observed at 1460 cm^−1^, while the peaks at 1275 and 1190 cm^−1^ are attributed to C–O stretching of acid groups. For the recycled products obtained via acidolysis and used to synthesize nylon, the spectra show a peak at 3300 cm^−1^, corresponding to NH_2_ functional groups, and peaks at 2930 and 2850 cm^−1^, attributed to methylene groups. Peaks centered at 1635 cm^−1^ and 1535 cm^−1^ are associated with C=O stretching of amide I and N–H bending of amide II, respectively, both characteristic of the nylon structure. No notable differences are observed between the synthesized nylon and nylon containing 10% recycled material.

Complementarily, in Figure 4b, the ^13^C NMR spectra of the synthesized nylon are presented. The peak at 173.5 ppm is attributed to the carbon atoms of the carbonyl groups. The peaks at 42.4 ppm and 36.0 ppm correspond to carbon atoms adjacent to NH_2_ groups and those near the carbonyl groups, respectively, while the peaks at 30.5 ppm and 25.0 ppm are assigned to methylene groups located in the middle of the nylon chains [28].

The XRD patterns of the synthesized nylon samples are presented in Figure 4c, where peaks between 2θ ≈ 20° and 24° are observed and associated with the (200) and (002/202) planes of the crystalline phase [29]. Moreover, the incorporation of RP does not produce significant changes in the crystallinity of nylon, indicating that the addition of recycled products does not affect the crystalline fraction of the polymer.

From the mechanical properties results presented in Table 2, it can be observed that the neat nylon sample presents a tensile strength of 1081.7 ± 180.0 kPa, while the recycled material reached 1791.1 ± 476.1 kPa. In a similar manner, the Young’s modulus increases from 40.2 MPa to 56.5 MPa (~41%), while the elongation at break decreases slightly from 3.5% to 2.9%, when recycled material is incorporated. This combination of increased stiffness and slightly reduced ductility is typical of semi-crystalline polymers undergoing thermal processing. During recycling, partial recrystallization or chain alignment can occur, increasing the rigidity of the material. At the same time, minor oxidative degradation or reduced chain mobility can slightly lower the ability of the material to deform before failure. Both virgin and recycled nylon remain brittle overall, with very low elongation values.

**Figure 5:**
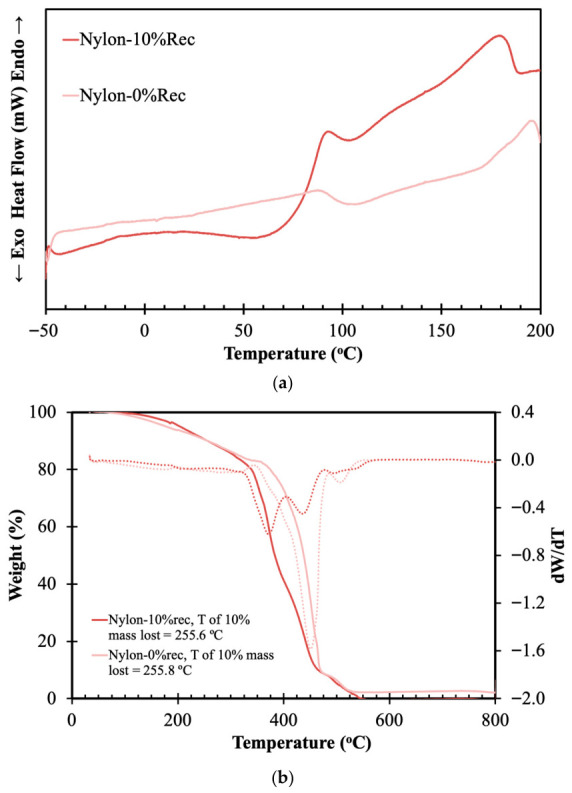
DSC (**a**) and TGA (**b**) of synthetized nylon and nylon containing 10% recycled material.

Next, the thermal behavior of nylon materials was determined, with the results presented in Figure 5.

Nylon is a semi-crystalline polymer characterized by strong intermolecular hydrogen bonding and its thermal behavior is highly sensitive to structural modifications. Figure 5a shows that the virgin nylon sample presents a relatively smooth thermal profile with a gradual increase in heat flow and a weak transition around 85–100 °C, corresponding to the glass transition and relaxation of amorphous regions. In contrast, the nylon sample containing 10% recycled fibers exhibits a more pronounced thermal event within the same temperature range, followed by a continuous increase in heat flow up to approximately 180 °C. A distinct high-temperature peak is also observed near 175–185 °C. The incorporation of recycled nylon likely introduces shorter polymer chains generated during previous thermal and mechanical processing. These shorter chains possess higher mobility and may reorganize more effectively into crystalline domains during heating, resulting in stronger recrystallization and melting-related transitions.

Analyzing the TGA results presented in Figure 5b, it can be seen that both samples exhibit an identical thermal degradation profile, characteristic of nylon [30]. The primary decomposition occurs between 350 °C and 500 °C, involving chain scission that breaks the polymer backbone into smaller volatile compounds. A closer examination of the TGA data further indicates that the incorporation of recycled material does not affect the thermal stability of the samples, as both exhibit a similar temperature for 10% mass loss.

### 4.3. Glycolysis of Polyester Fibers

**Figure 6:**
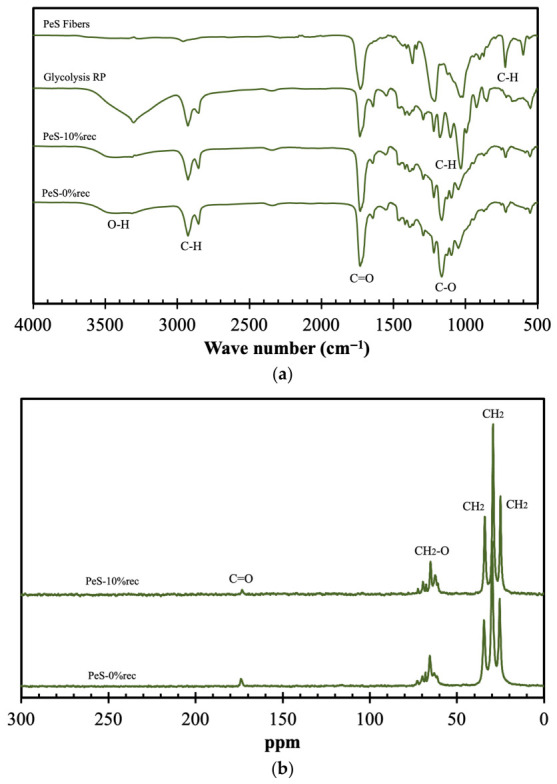

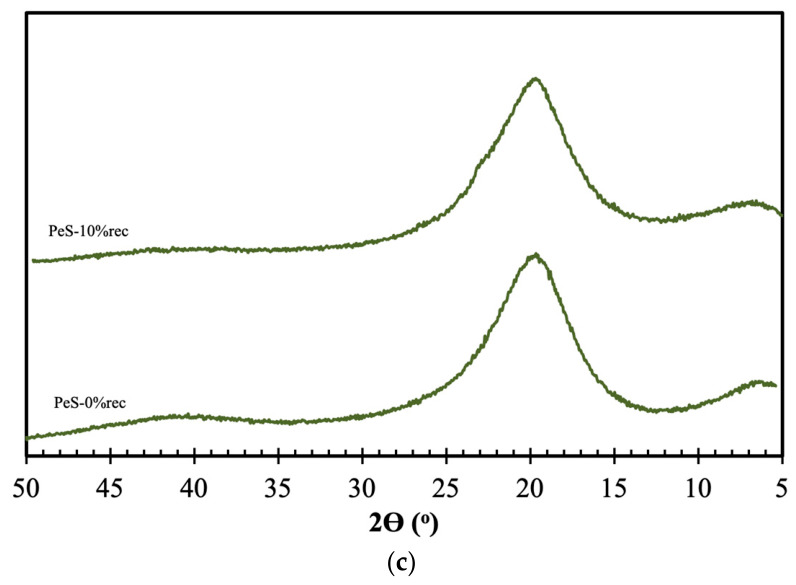
Spectroscopic analyses of original PeS fibers, products obtained via glycolysis of PET both synthetized PeS and PeS containing 10% recycled material: FTIR (**a**), ^13^C CPMAS (**b**) and XRD (**c**).

The next selective valorization step consisted in the glycolysis of the PeS fibers, with the spectroscopic analyses of the ensuing products presented in Figure 6.

In Figure 6a, the characteristic peaks of PET are observed, including the peak at 1710 cm^−1^ attributed to C=O stretching, the region between 1000 and 1240 cm^−1^, corresponding to C-O moieties and the peak around 725 cm^−1^ assigned to aromatic C–H bending [31]. In addition to these peaks, the recycled product obtained via glycolysis shows characteristic signals of the solvents used (glycerol and PEG 400), including the O–H stretching at 3447 cm^−1^, the CH_2_ stretching at 2865 cm^−1^ and the symmetric C–O–C stretching at 1095 cm^−1^. The recycled product was subsequently used to synthesize new PeS, which exhibit an FTIR profile identical to the neat counterpart. In these spectra, the broad band between 3200 and 3600 cm^−1^ is related to O–H stretching. The region between 1650 and 1800 cm^−1^ corresponds to C=O stretching from esters and carboxylic acid groups, while the region from 1000 to 1100 cm^−1^ is attributed to primary and secondary alcohol C–O stretching (1052 and 1076 cm^−1^, respectively) [32].

Regarding the ^13^C CPMAS spectra presented in Figure 6b, the PET samples exhibit peaks in the 25–35 ppm range, primarily arising from CH_2_ groups. The peaks at 60 and 70 ppm are attributed to CH_2_–COO and CH_2_–O groups, respectively, while the peak at 164 ppm corresponds to the C=O groups [33,34]. As in the case of FTIR spectra, no differences are observed between the ^13^C CPMAS spectra of the synthesized PS and the PS containing 10% recycled material.

Similarly, from Figure 6c, identical spectra can be observed. The XRD pattern of both PeS materials exhibits a broad peak in the 15–25° range, confirming the predominantly amorphous structure of the samples. It should be noted that the recycled products derived from PET textile fibers are typically crystalline polymers (reflections at 2θ ≈ 26–29°, 37° 43°, attributed to the (100), (101) (200) crystallographic planes, respectively [35]). The absence of these peaks indicates that the incorporation of recycled PET did not significantly alter the amorphous nature of the PeS matrix [36].

With regard to the mechanical properties presented in Table 2, tensile strength values of 2.8 ± 0.1 kPa for the neat material and 3.7 ± 0.7 kPa after recycling can be observed, while the Young’s modulus remains low and essentially unchanged (324.0 kPa vs. 344.0 kPa). Additionally, the elongation at break increases from 60.5% to 103.2%, almost doubling, when recycled products are incorporated. Glycolysis leads to chain scission, generating low-molecular-weight oligomers and monomers with a hydroxyl end group. These shorter and more flexible chain segments contribute to increased chain mobility and reduced intermolecular interactions [37,38]. As a result, the material exhibits a more ductile behavior, increasing the elongation at break.

**Figure 7:**
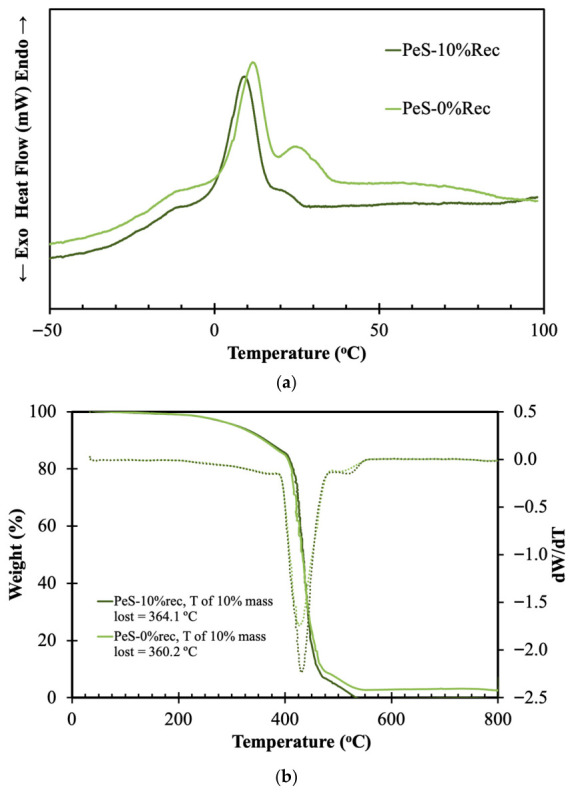
DSC (**a**) and TGA (**b**) of synthetized PeS and PeS containing 10% recycled material.

Lastly, the DSC and TGA of the synthetized PeS samples were evaluated, with the results presented in Figure 7a and Figure 7b, respectively.

The PeS thermograms presented in Figure 7a show sharp and well-defined thermal peaks, exhibiting a strong endothermic peak near 10–15 °C for the virgin PeS sample, followed by a secondary transition around 20–30 °C and a broad relaxation region extending toward higher temperatures. In comparison, the PeS sample containing 10% recycled fibers presents lower peak intensity and broader thermal transitions. During recycling, repeated thermal exposure and mechanical stress can promote chain degradation and structural disorder. Consequently, the recycled PeS sample exhibits increased amorphous character.

From the TGA results presented in Figure 7b, it can be observed that both samples exhibit an identical single-step degradation, occurring between 370 °C and 450 °C, caused by the cleavage of the ester bonds in the polymer’s main chain [39,40]. Furthermore, both samples reach 10% mass loss at similar temperatures, indicating comparable thermal stability at the onset of degradation. This suggests that the incorporation of recycled PET-derived components into the PeS structure does not significantly affect the thermal degradation behavior, which remains primarily governed by the stability of the ester linkages along the polymer backbone.

### 4.4. Acetylation of Cellulose Fibers

**Figure 8:**
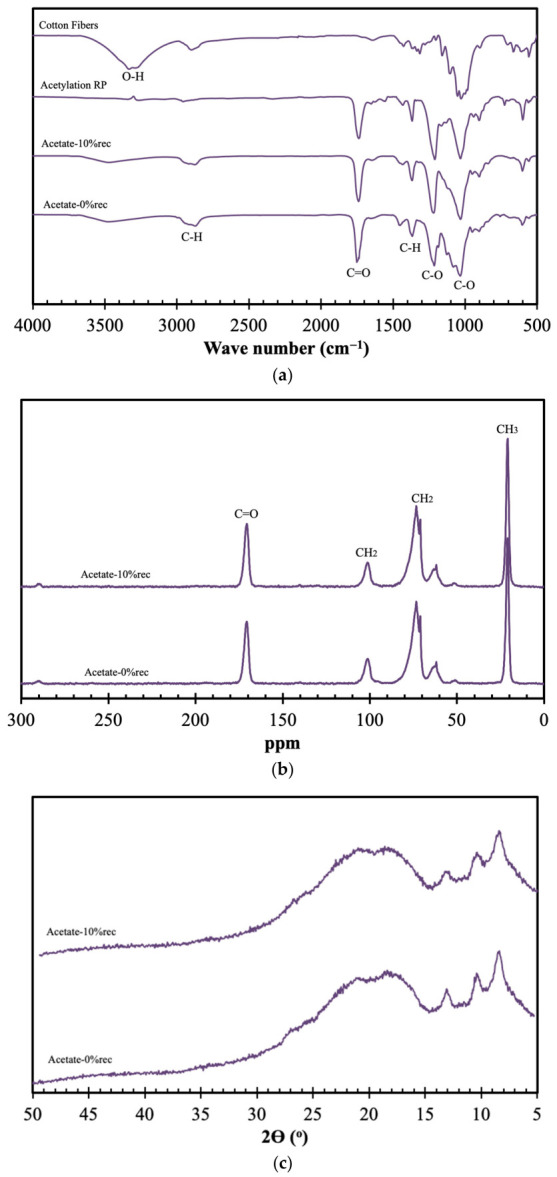
Spectroscopic analyses of original cotton fibers, products obtained via acetylation of cotton, both virgin acetate and acetate containing 10% recycled material: FTIR (**a**), ^13^C CPMAS (**b**) and XRD (**c**).

The final valorization step of the valorization of a mixture of fibers consisted in the acetylation of cotton materials. In Figure 8, the spectroscopic analyses of the ensuing materials are presented.

As illustrated in Figure 8a, the FTIR spectrum of cotton fiber shows a broad band between 3100 and 3600 cm^−1^, corresponding to O–H stretching, as well as peaks at 2865 cm^−1^ due to CH_2_ stretching and a broad peak at 1030 cm^−1^ attributed to C–O vibrations of cellulose moieties. For the acetylated sample, clear evidence of acetylation is observed, with three characteristic ester bands appearing at 1740 cm^−1^ (C=O stretching of the ester), 1370 cm^−1^ [C–H present in –O(C=O)–CH_3_] and 1230 cm^−1^ (C–O stretching of the acetyl group) [41]. Additionally, the absence of the O–H stretching band at 3337 cm^−1^ further confirms the successful acetylation. Regarding the incorporation of this product into cellulose acetate, it can be observed that both the neat material and the incorporated counterpart exhibit identical FTIR profiles, indicating a strong similarity between the samples.

The FTIR results are in agreement in the ^13^C CPMAS results presented in Figure 8b, where identical spectra are observed. In Figure 8b, the typical ^13^C CPMAS signals of cellulose acetate are observed at 21 ppm (CH_3_), C6: 62–65 ppm, C2–C3–C4–C5 cluster: 70–90 ppm, C1: 105 ppm and 171 ppm (C=O) [42].

Likewise, the XRD spectrum presented in Figure 8c of the sample with 10% recycled material closely matches that of the neat counterpart, reflecting a high degree of similarity between the samples. Both diffractograms exhibit a predominantly broad halo in the 2θ range of approximately 15–25°, which is characteristic of the amorphous structure of cellulose acetate. This broad diffraction profile indicates the absence of long-range crystalline order, confirming that the material is mainly amorphous. Additionally, low-angle reflections around 2θ ≈ 8°, 10°, 13° can be observed, which are also typically associated with cellulose acetate [43,44].

From Table 2, it can be observed that the cellulose acetate sample incorporated with recycled products exhibits a slightly lower stiffness compared to the neat sample. Cellulose acetate presents a tensile strength of 5662.2 ± 1092.2 kPa, while the recycled material counterpart exhibited a value of 3016.0 ± 650.4 kPa. Furthermore, the Young’s modulus decreased from 179.2 MPa to 169.2 MPa (approximately 6%). At the same time, the elongation at break decreases from 7.9% to 5.3% (approximately 33%), indicating a reduction in ductility and an increase in brittle behavior. This suggests that the incorporation of recycled components introduces minor structural heterogeneities within the cellulose acetate matrix, which may act as stress concentration sites and restrict molecular mobility. As a consequence, the effect on ductility is more pronounced than on stiffness, reflecting a limited but measurable disruption of the polymer’s deformation mechanism.

**Figure 9:**
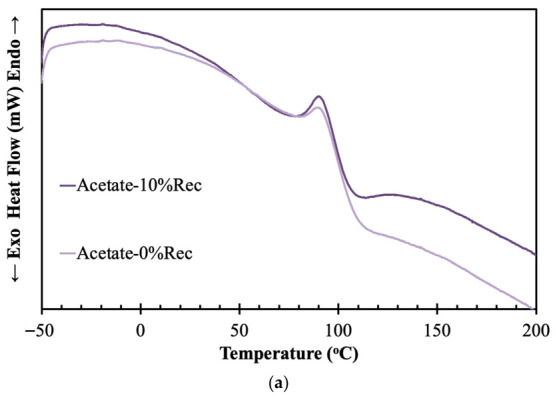

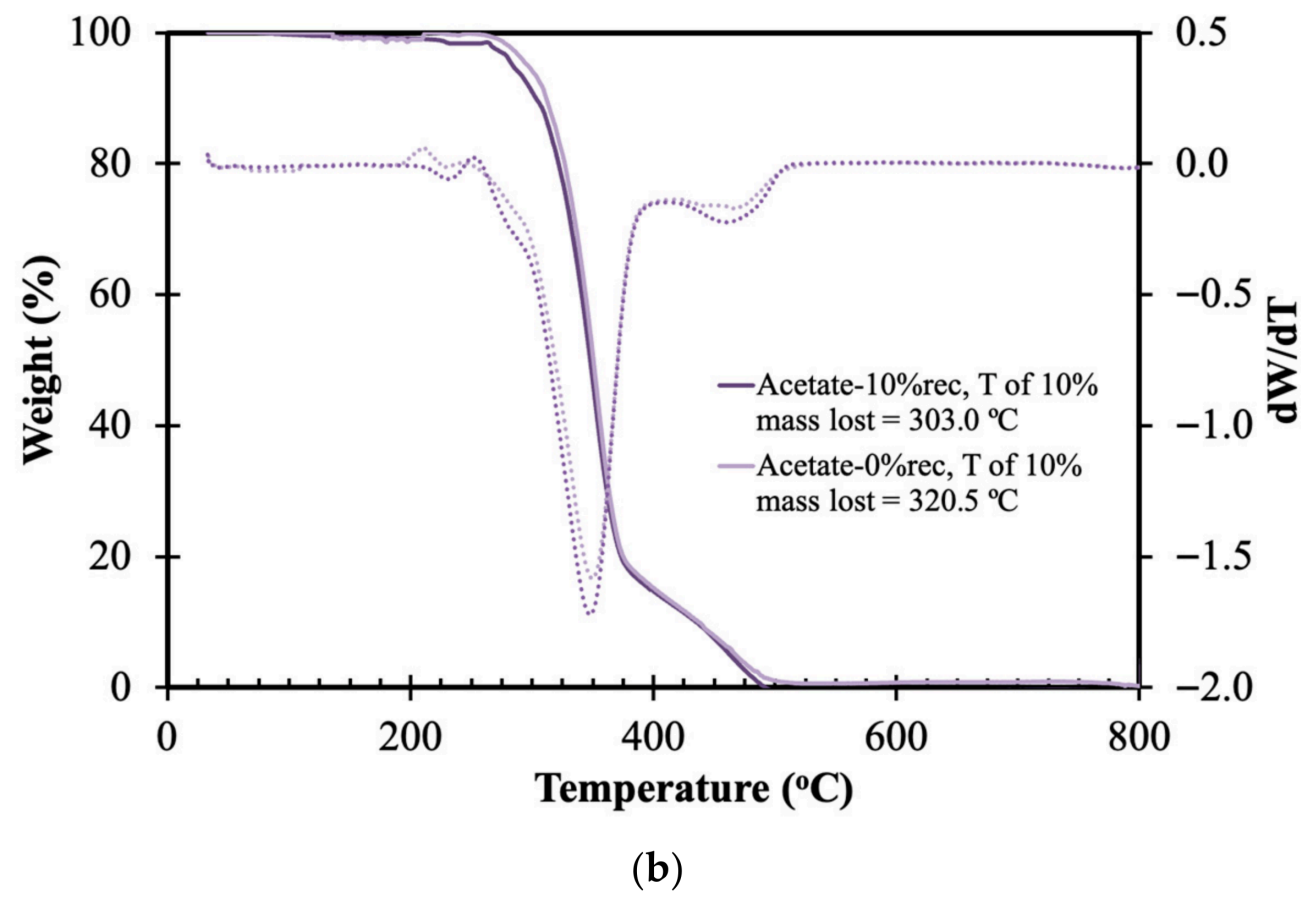
DSC (**a**) and TGA (**b**) of virgin acetate and acetate containing 10% recycled material.

Lastly, the thermal analysis results of the cellulose acetate samples are presented in Figure 9.

The cellulose acetate thermograms depicted in Figure 9a reflect the predominantly amorphous nature of cellulose acetate fibers. Both acetate samples display broad thermal transitions rather than sharp melting peaks. The virgin acetate sample shows a gradual decrease in heat flow with a noticeable transition around 90–100 °C, which is mainly associated with the glass transition and molecular relaxation processes. After this transition, the curve decreases continuously with increasing temperature, indicating progressive softening of the polymer structure. The acetate sample containing 10% recycled fibers exhibits a similar overall thermal profile, although it maintains slightly higher heat-flow values after the transition region. This behavior suggests that recycling induces only modest structural changes. The slightly improved thermal response of the recycled sample may be related to localized chain rearrangement or increased intermolecular interactions generated during recycling, indicating good compatibility between virgin and recycled fibers.

From Figure 9b, it can be observed that both the neat acetate and the acetate containing 10% recycled material exhibit an identical thermal degradation profile. The weight loss occurring between 300 °C and 400 °C, corresponding to the main degradation region, is attributed to the deacetylation process of cellulose acetate, as described by Fenzo et al. [45]. Additionally, the sample containing recycled material shows slightly higher thermal stability. This effect may be explained by the fact that the thermal degradation of cellulose-based materials can act as a radical scavenger in radical aging reactions, due to the presence of significant amounts of polyphenolic compounds, mainly lignin and tannins [46].

Overall, the feasibility of selectively valorizing mixed textile waste through polymer-specific chemical recycling routes was demonstrated, enabling the recovery and reprocessing of individual fiber types without cross-contamination. The incorporation of 10 wt% recycled products into new polymer matrices generally preserved chemical structure and thermal stability, while mechanical properties varied depending on the polymer and recycling pathway.

## 5. Conclusions

This study demonstrates the feasibility of a selective recycling approach for mixed textile waste, enabling recovery and reprocessing of acrylic, nylon, PeS and cellulose-derived fractions. Spectroscopy analyses confirmed that all recycled products retained their main structural features after reprocessing, with no significant differences observed between virgin and 10 wt% recycled-containing materials. In acrylic systems, partial modification of nitrile groups was evidenced by the disappearance of the 2240 cm^−1^ band, indicating possible hydrolysis during dissolution–precipitation, yet thermal analysis showed that the incorporation of recycled fractions did not significantly compromise stability. In fact, degradation temperatures remained essentially unchanged across all systems: acrylic (10% mass loss at 235 °C for virgin material), nylon (350–500 °C), PeS (370–450 °C) and cellulose acetate (300–400 °C), with only minor improvements observed in some recycled-containing formulations. Regarding the mechanical properties, the observed variations depended on the polymer chemistry and the recycling route used. Acrylic samples had an 11% reduction in modulus and a 25% decrease in ductility, while nylon showed a 41% increase in stiffness with only a slight reduction in elongation (3.5% to 2.9%). PeS exhibited a dramatic increase in elongation from 60.5% to 103.2%, indicating enhanced ductility after glycolysis-derived incorporation. Cellulose acetate showed modest stiffening loss (−6%) and a more pronounced reduction in elongation (−33%). Overall, incorporating 10 wt% recycled material generally preserves thermal and chemical integrity while inducing mechanical property varies depending on polymer type. These results confirm that controlled chemical recycling routes enable effective upcycling of mixed textile waste streams into compatible polymer systems, supporting circular economy strategies for textile-to-polymer valorization.

## Figures and Tables

**Table 1:** Valorization methods used for different textile fiber types (“Yes” indicates that the valorization method was successful, whereas “No” indicates that the method was not successful).

	Dissolution–Precipitation	Acidolysis	Glycolysis	Acetylation
Acrylic	Yes	No	No	No
Nylon	No	Yes	Yes	No
Polyester	No	No	Yes	No
Cotton	No	No	No	Yes

**Table 2:** Mechanical properties of materials produced.

Sample	Tensile Strength (kPa)			Young Modulus (MPa)			Elongation at Break (%)		
Acrylic	6328.9	±	225.4	532.1	±	20.7	50.6	±	4.0
Acrilic rec	3756.8	±	351.1	473.7	±	23.1	37.8	±	1.6
Nylon	1081.7	±	180.0	40.2	±	4.3	3.5	±	0.7
Nylon rec	1791.1	±	476.1	56.5	±	2.3	2.9	±	0.8
PeS	2.8 *	±	0.1 *	324.0	±	35.1	60.5	±	6.0
PeS rec	3.7 *	±	0.7 *	344.0	±	45.1	103.2	±	11.2
Acetate	5662.2	±	1092.2	179.2	±	7.6	7.9	±	1.1
Acetate rec	3016.0	±	650.4	169.2	±	11.3	5.3	±	0.8

* Tensile strength in kPa.

## Data Availability

The datasets presented in this article are not readily available because due to privacy. Requests to access the datasets should be directed to the corresponding author.
